# Baseline isotopic variability in plants and animals and implications for the reconstruction of human diet in 1 st century AD Pompeii

**DOI:** 10.1038/s41598-025-12156-7

**Published:** 2025-08-03

**Authors:** Silvia Soncin, Valeria Amoretti, Chiara Comegna, Chiara Assunta Corbino, Noemi Mantile, Simona Altieri, Maria Rosa Di Cicco, Valentina Giacometti, Jan Bakker, Marina Caso, Angela Trentacoste, Steven Ellis, Mary Anne Tafuri, Gabriel Zuchtriegel, Oliver Edward Craig, Carmine Lubritto

**Affiliations:** 1 Department of Environmental Biology and Mediterranean bioArchaeological Research Advances (MAReA) Centre, Sapienza Università Di Roma, Rome, Italy; 2 Pompeii Archaeological Park, Pompeii, Naples, Italy; 3 Department of Environmental Biological and Pharmaceutical Science and Technology and Mediterranean bioArchaeological Research Advances (MAReA) Centre, University of Campania “L. Vanvitelli”, Caserta, Italy; 4 ACASA, University of Amsterdam, Amsterdam, The Netherlands; 5 Herculaneum Archaeological Park, Ercolano, Naples, Italy; 6 The British School at Rome, Rome, Italy; 7 Department of Classics, University of Cincinnati, Cincinnati, OH U.S.; 8 Department of Archaeology, BioArCh, University of York, York, UK; 9 CHNet INFN Sez. Napoli, Naples, Italy

**Keywords:** Stable isotopes, Pompeii, Roman economy, Roman diet, Agriculture, Husbandry, Anthropology, Archaeology, Biochemistry, Biogeochemistry, Environmental sciences

## Abstract

While Pompeii has long captured the imagination with its history and tragic end, recent efforts have shifted towards unveiling everyday lifeways. Our study seeks to explore agricultural and husbandry practices in Pompeii, aiming to explore isotopic variability of different food categories available to the Romans within this unique “snapshot” scenario. To do so, we deploy stable carbon and nitrogen isotope analysis of plants and animals. Our findings suggest a diversity of practices, with isotopic variation in C_3_ cereals and legumes pointing to the use of a greater variety of cultivation techniques compared to arboreal crops. We highlight distinct management regimes utilised for different animal species and we uncover a spectrum of aquatic environments, indicative of diversity of fishing practices. These findings provide direct support of archaeological evidence and textual interpretations of Roman food systems in Pompeii. However, our dataset also reveals the limitations of bulk isotope approaches in detecting this dietary diversity when we use it to interpret the local human diet through mixing models. Together, our results show that a broad and well-contextualised isotopic baseline can help us understanding ancient food systems, while also revealing the challenges of disentangling dietary complexity using bulk stable isotope data alone.

## Introduction

**Fig. 1:**
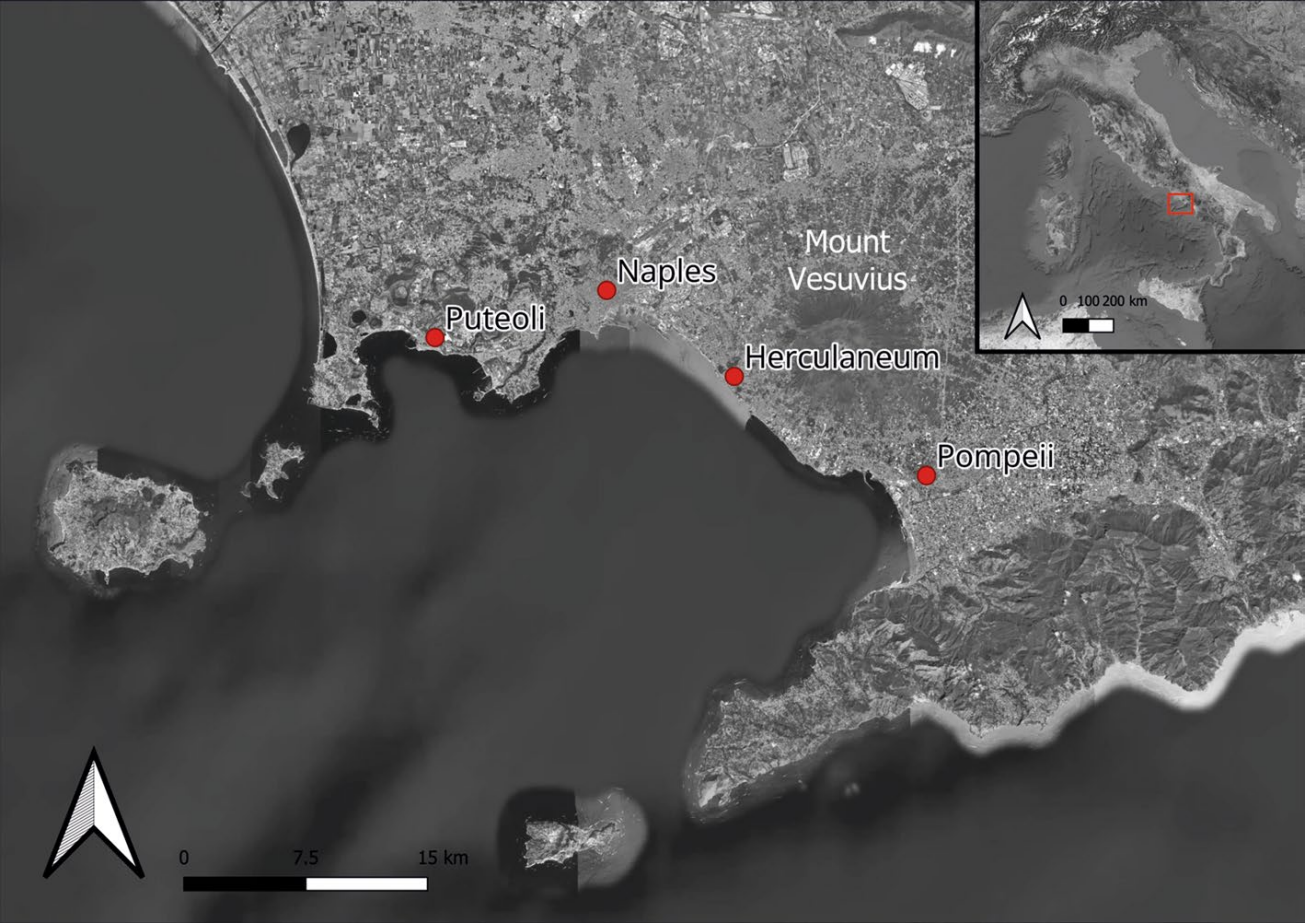
Map of the Gulf of Naples with location of Pompeii and other archaeological sites and landmarks using QGIS version 3.34.2, base map ESRI Satellite.

The World Heritage Site of Pompeii is the second most visited archaeological site in Italy, and one of the most visited in the world, counting approximately 4 million visitors in 2023 (https://pompeiisites.org/parco-archeologico-di-pompei/dati-visitatori/). The fame of Pompeii among the general public is mainly related to the Mount Vesuvius eruption in AD 79. Beyond the aspects of the human tragedy and mainly due to its remarkable preservation, Pompeii represents a unique opportunity to investigate various aspects of everyday Roman lifeways in a particularly fortunate corner of the Empire: the Gulf of Naples and the area of *Campania* more in general (Fig. 1).

The fertile soil and the high-water availability of this region made it ideal for human settlements long before the Roman domination^1^. By the second century BC, the Bay of Naples had reached a population density and an intensity of land use with few parallels in the ancient world^2^. The area benefited from Mediterranean connections through the sheltered harbour of Puteoli, which remained for centuries the main harbour of the Roman Empire in the Italian peninsula. Until Trajan built the new harbour of Rome, Puteoli was the main offloading point for goods coming from the Roman provinces^3^.

Despite the fertile Sarno River floodplain, Pompeii was likely not self-sufficient and depended on imports and nearby production to meet nutritional needs. Certainly, there is evidence for local cultivation. Ancient sources report that four varieties of grapes (*Vitis vinifera*) were cultivated along the Vesuvian slopes and that two-thirds of Mount Vesuvius were dedicated to vineyards, making this area a “wine district” of the Empire^4^. Figs (*Ficus carica*) are ubiquitous in Pompeii as well as Herculaneum, a nearby Roman town also destroyed by the eruption, suggesting a local cultivation of fig trees (Plin. *HN*, XV, 71; Cato, *Agr*, VIII,1). Broad beans (*Vicia faba*) are also very common in Pompeii, likely reflecting specialised local cultivation not only for consumption, but also for agricultural purposes such as soil enrichment^5^. Tree crops, including walnut (*Juglans regia*), are attested by the pollen record^6^. Although olive oil was produced locally^6^, production was probably insufficient to support urban needs in the area. As for grains, both written and archaeological evidence suggest a lack of large-scale production in the area; however, the presence of pollen belonging to some species of cereals confirms that some production of grains existed in the Sarno River floodplain^6^. Given the estimated importance of cereals in the Roman diet (up to 70% of total calories considering the quantity of grains distributed during the *frumentationes*)^7^, much of the supply was likely imported from nearby regions or the Provinces^4^. Historical accounts also confirm large shipments of grain and pulses from Egypt to Puteoli for local distribution^8^.

As for domestic animals, the abundance of Cichorieae plants and the presence of charcoal and coprophilous fungal spores suggest widespread use of the floodplain as pasture^6^. The importance of fish for the Romans is also well attested. Exploitation included trapping in lagoons and farming species such as gilt-head bream (*Sparus aurata*)^9^. The Mediterranean is also ideal for the fishing of pelagic migratory fish, notably tuna but also mackerel and bonito, large schools of which enter the warm waters of the Mediterranean from the Atlantic to reproduce^9^*.* A remarkable range of fish and shellfish species from the Cardo V sewer in Herculaneum attests their presence in the Gulf of Naples^10^. These species exhibit diverse behavioural characteristics, with some living nearshore, others offshore, and some others in the Sarno estuary, requiring different fishing tools and strategies^8^.

Although historical and archaeological evidence has provided significant insights into the ancient economy of Pompeii, much of this knowledge relies on indirect proxies, such as literary sources, or remains fragmentary, making it difficult to determine whether certain findings reflect broader economic patterns or are context-specific (e.g., the remains from the Cardo V sewer in Herculaneum). A direct evaluation of Pompeii’s economic and dietary realities is still lacking. Here we present and discuss stable carbon and nitrogen isotope results from faunal and botanical remains from Pompeii and surroundings. The unique nature of this dataset, capturing a rare snapshot of food consumption within a narrow time frame—a few decades or less in the 1st century AD—offers an exceptional opportunity to investigate isotopic variability in local food webs without the chronological uncertainties typically associated with archaeological isotope studies. Furthermore, we use this temporally constrained context to evaluate the potential and limitations of the bulk stable isotope approach in reconstructing human diets, examining whether this method can effectively capture dietary diversity in a society as complex as Pompeii.

## Results and discussion

Data and details of the samples analysed are reported in Supplementary Table 1. Sampling strategy, contextual details and methods are discussed in Supporting Information. Results from previous publications have also been included in the analysis^11,12^ (Supplementary Table 1). All plant samples were included in the study as their isotopic signatures showed no correlation with their carbon and nitrogen content, which would point towards diagenesis (Supplementary Fig. 1)^13^. However, one olive stone sample (*Olea europaea*, PPOE2) was not included due to a significantly higher C:N ratio. We excluded 1 sample (PAEq1) from the faunal group due to collagen failing to meet commonly accepted quality criteria^14^. While certain categories are represented by few samples, the overall dataset is substantial, particularly when broader analytical groupings are considered. This reflects both the exceptional preservation of the assemblage, and a strategy designed to maximise interpretative potential across categories. Table 1 presents descriptive statistics for these categories.

**Table 1:** Descriptive statistics for botanical and faunal samples from Pompeii and surroundings. This also includes previously published material^11,12^.

	**n**	**δ**^**13**^**C (‰)**						**δ**^**15**^**N (‰)**					
		**Mean**	**Median**	**1SD**	**CIs (95%)**	**Min**	**Max**	**Mean**	**Median**	**1SD**	**CIs (95%)**	**Min**	**Max**
**Botanical samples**													
Bread	3	−21.7	−21.7	0.1	(−21.9, −21.5)	−21.8	−21.6	6.5	7.1	4.8	(−5.4, 18.3)	1.4	10.9
C_3_ Cereal	14	−24.1	−23.5	2.7	(−25.7, −22.5)	−31.4	−20.6	4.9	4.7	3.2	(3.0, 6.7)	0.8	11.9
C_3_ Arboreal Crop	11	−25.6	−25.4	1.0	(−26.3, −24.9)	−28.0	−24.1	3.4	2.8	3.0	(1.3, 5.4)	−0.1	8.1
C_4_ Cereal	2	−10.7	−10.7	0.6	(−16.4, −4.9)	−11.1	−10.2	2.0	2.0	0.2	(0.0, 3.9)	1.8	2.1
Legume	14	−24.0	−24.6	2.3	(−25.3, −22.7)	−27.0	−20.7	1.1	0.8	1.3	(0.4, 1.9)	−0.5	5.0
**Faunal samples**													
*Terrestrial herbivores*	15	−20.5	−20.6	0.6	(−20.8, −20.1)	−21.3	−18.8	4.1	3.7	1.5	(3.3, 4.9)	2.0	6.8
*Bos taurus*	3	−20.3	−20.3	0.2	(−20.7, −20.0)	−20.5	−20.2	4.8	4.3	1.8	(0.5, 9.2)	3.4	6.8
*Capra hircus*	4	−19.9	−20.0	1.2	(−20.5, −18.3)	−21.3	−18.5	3.4	3.4	0.7	(1.6, 4.8)	2.7	4.2
*Equus* sp.	4	−20.2	−20.6	1.0	(−21.7, −18.7)	−20.9	−18.8	3.1	3.2	1.0	(1.5, 4.7)	2.0	4.2
*Ovis aries*	2	−21.1	−21.1	0.4	(−24.2, −17.9)	−21.3	−20.8	6.5	6.5	0.1	(5.2, 7.8)	6.4	6.6
Terrestrial omnivores	24	−19.1	−19.6	2.1	(−20.0, −18.2)	−21.7	−13.4	5.7	5.6	2.1	(4.8, 6.6)	2.4	9.3
*Canis familiaris*	3	−18.9	−18.9	0.3	(−19.6, −18.2)	−19.2	−18.6	8.7	8.7	0.2	(8.2, 9.2)	8.5	8.9
Columbidae	2	−17.6	−17.6	5.9	(−70.3, 35.2)	−21.7	−13.4	5.5	5.5	0.1	(4.2, 6.8)	5.4	5.6
*Gallus domesticus*	5	−16.8	−16.8	1.0	(−18.0, −15.6)	−18.2	−15.6	5.9	5.6	0.9	(4.8, 6.9)	4.8	7.0
*Sus scrofa*	14	−20.4	−20.7	0.8	(−20.9, −20.0)	−21.4	−18.5	4.7	3.9	1.9	(3.6, 5.8)	2.4	7.9
Fish	15	−12.7	−13.1	1.5	(−13.5, −11.8)	−14.3	−9.4	8.4	8.4	1.6	(7.5, 9.2)	5.7	11.8
Scombridae	6	−13.5	−13.4	0.6	(−14.1, −12.9)	−14.3	−12.8	7.9	7.9	1.0	(6.9, 8.9)	6.5	9.1
Sparidae	5	−11.7	−12.6	2.0	(−14.2, −9.3)	−13.9	−9.4	7.7	7.4	1.6	(5.7, 9.7)	5.7	9.5

### The botanical assemblage from the territories of the eruption

The plant assemblage includes charred specimens sourced from old and new excavations from Pompeii and other contexts impacted by the AD 79 eruption. Therefore, variations in their stable carbon and nitrogen isotope values more likely reflect differences in their growing conditions rather than fluctuations due to changes in climatic or environmental conditions within the same location through time. While some samples might have been stored for longer periods of time after harvest, it is thought that grains and beans were predominantly consumed annually of perhaps over a year or two, there being little economic incentive for longer-term storage^15^.

**Fig. 2:**
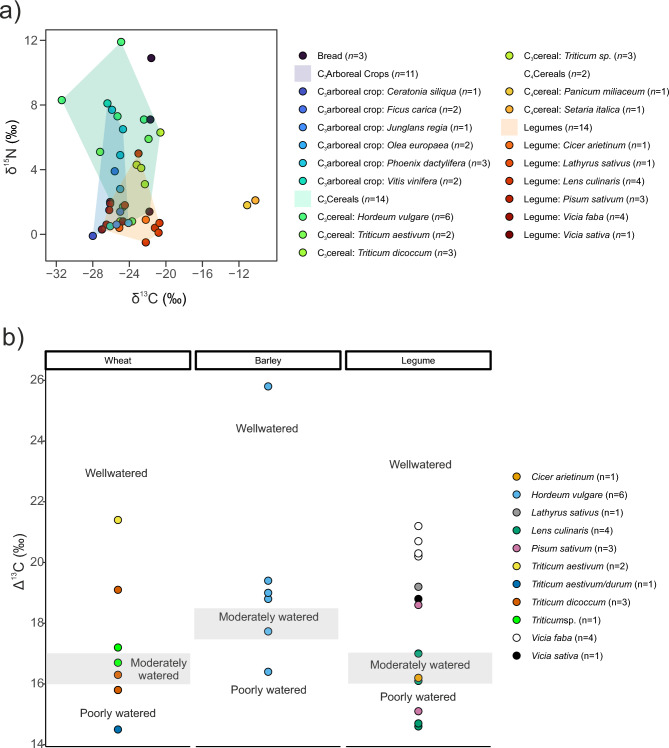
δ^13^C, δ^15^N (**a**) and Δ^13^C (**b**) values of plant material from Pompeii and its surroundings analysed in this study. (**a**) polygons represent the convex hull areas that cover the distribution of values of each category^23^. (**b**) grey bands represent regions indicative of moderately watered growing conditions^29^.

We have divided the botanical remains into groups representing different food categories. These are bread samples (*n* = 3), C_3_ cereals (*n* = 14), C_3_ arboreal crops (*n* = 11, which includes fruits and nuts, as well as one sample of carob—botanically a legume, but grouped here due to the likely consumption of the whole pod for its culinary properties, including thickening and sweetening uses; see Pliny: *HN*, XXIII, 79; Cato: *Agr*, XCVI; Apicius: *De Re Coq*, IV, II, 14), C_4_ cereals (*n* = 2), legumes (*n* = 14) and olive oil (*n* = 1). C_3_ cereals, legumes and C_3_ arboreal crops share a similar distribution of stable carbon isotope values, consistent with the three categories belonging to the same photosynthetic group (Table 1; Fig. 2a). However, C_3_ cereals are more variable in their stable carbon isotope values compared to C_3_ arboreal crops and the majority of C_3_ cereals and legumes exhibit higher values compared to C_3_ arboreal crops (Fig. 2a). Based on archaeological and historical records, it’s reasonable to assume that the C_3_ arboreal crops considered here are predominantly from the town’s hinterland. Ancient sources often refer to local products, an example being the “fig of Herculaneum” mentioned by Pliny (*HN*, XV, 71) and Cato (*Agr*, VIII, 1). One exception is that of dates (*Phoenix dactylifera*), which are probably imported from distant regions^16^. Interestingly, the three dates analysed here exhibit higher δ^15^N values compared to other C_3_ arboreal crops (Supplementary Table 1), which may reflect a more arid area of origin^17,18^. When dates (*Phoenix dactylifera*) are removed from the analysis, the mean δ13C value for C₃ arboreal crops is −25.6‰ [CI: −26.3, −24.9], compared to −24.1‰ [CI: −25.7, −22.5] for C₃ cereals. For δ^15^N, arboreal crops average 3.4‰ [CI: 1.3, 5.4], and cereals 4.9‰ [CI: 3.0, 6.7]. These differences are statistically significant (Wilcoxon rank-sum test: W = 90, p-value = 0.02208 and W = 88.5, p-value = 0.02887, respectively), with average shifts of approximately 1.5‰ in both δ13C and δ15N value.

C_3_ cereals produced the largest range of stable isotope values among all the plant groups. The varied δ^13^C and δ^15^N values of C_3_ cereals reflect highly varied environmental, climatic and water availability conditions^19–21^ (Table 1;Fig. 2a). Additionally, we cannot exclude that the observed δ^15^N values could be indicative of different degrees of manure usage^22,23^.

Legumes exhibit δ^13^C values typical of C_3_ plants (Table 1;Fig. 2a). However, their wide range of values could demonstrate diverse growing and environmental conditions. In contrast, their δ^15^N values are generally lower—a typical trait of legumes due to their nitrogen-fixing capacity—and show a more tightly clustered distribution compared to those observed in C_3_ plants and C_3_ cereals, also typical of this group since legumes have been found to demonstrate less sensitivity to variations in manure application rates, with discernible effects at the isotopic level only under extremely high manuring conditions^23^.

The two samples representing the C_4_ cereal group exhibit consistent δ^13^C and δ^15^N values (Table 1;Fig. 2a). Their stable carbon isotope values are characteristic of this specific photosynthetic group^24,25^, while their stable nitrogen isotope values suggest a minimal input of manure^26^.

The three bread samples included in this study display δ^13^C values consistent with a C_3_ origin, aligning with the values observed in C_3_ cereals, C_3_ plants and legumes, although among the most ^13^C-enriched ones. Conversely, their δ^15^N values are more variable (Table 1;Fig. 2a).

Next, we converted the δ^13^C values of barley, legume and wheat samples in Δ^13^C values^27^ using the values of atmospheric CO_2_ for the periods 10 and 189 AD^28^ (accessed from this link: https://data.mendeley.com/datasets/btwpwh8292/3. Table 2;Fig. 2b). This conversion enabled us to utilise reference experimental values for assessing the water conditions of wheat, barley, and legumes^29^. Barley appears to be predominantly well-watered, while wheat is primarily moderately watered, indicating potentially drier growing conditions for wheat (Table 2**; **Fig. 2b). However, this interpretation is approached with caution due to the limited number of samples.

**Table 2:** Descriptive statistics of δ^13^C, δ^15^N and Δ^13^C of barley, legume and wheat samples from Pompeii and surroundings.

		**δ**^**13**^**C (‰)**						**Δ**^**13**^**C (‰)**						**δ**^**15**^**N (‰)**					
	***n***	**Mean**	**Median**	**1SD**	**CIs (95%)**	**Min**	**Max**	**Mean**	**Median**	**1SD**	**CIs (95%)**	**Min**	**Max**	**Mean**	**Median**	**1SD**	**CIs (95%)**	**Min**	**Max**
Barley	6	−25.4	−24.8	3.1	(−28.7, −22.1)	−31.4	−22.4	19.5	18.9	3.3	(16.1, 22.9)	16.4	25.8	6.2	7.2	4.2	(1.8, 10.6)	0.8	11.9
Legume	14	−24.0	−24.6	2.3	(−25.3, −22.7)	−27.0	−20.7	18.1	18.7	2.4	(16.7, 19.4)	14.6	21.2	1.1	0.8	1.3	(0.4, 1.9)	−0.5	5.0
Wheat	8	−23.1	−22.5	2.1	(−24.8, −21.3)	−27.2	−20.6	17.1	16.5	2.2	(15.3, 18.9)	14.5	21.4	3.9	4.2	2.0	(2.2, 5.5)	0.8	6.3

The Δ^13^C values of legumes show wide variation (Table 2**; **Fig. 2b). Lentils consistently demonstrated poor-to-moderate water conditions, whereas broad beans consistently fell within the well-watered region (Student’s *t*-test: t = 8.0379, df = 6, p-value = 0.0001982). As pointed out in the introduction, broad beans may be local, while other pulses might have been imported, as is also inferred from the large quantities of broad beans recovered from the territories of the eruption compared to lentils^30^.

The higher degree of variation in isotopic signatures among C_3_ cereals and legumes compared to C_3_ arboreal crops suggests multiple growing conditions and environments. Differences in harvest timing and species-specific physiological traits may also contribute to this isotopic variation^21,29^. Considering the pivotal role of C_3_ cereals and legumes in the Roman diet^7^, it is not surprising to observe such differentiation when compared to C_3_ arboreal crops. This diversity is indicative of varied agricultural techniques, potentially linked to a mixture of local and non-local sourcing, and even different trade networks, although stable carbon and nitrogen isotopes cannot definitively pinpoint the geographic origins of plants.

### The faunal assemblage from Pompeii

In this paragraph we integrate the faunal samples analysed for this paper with those previously published from Pompeii^11,12^. All these samples belong either to the levels of the eruptions or more broadly to the 1st century AD.

**Fig. 3:**
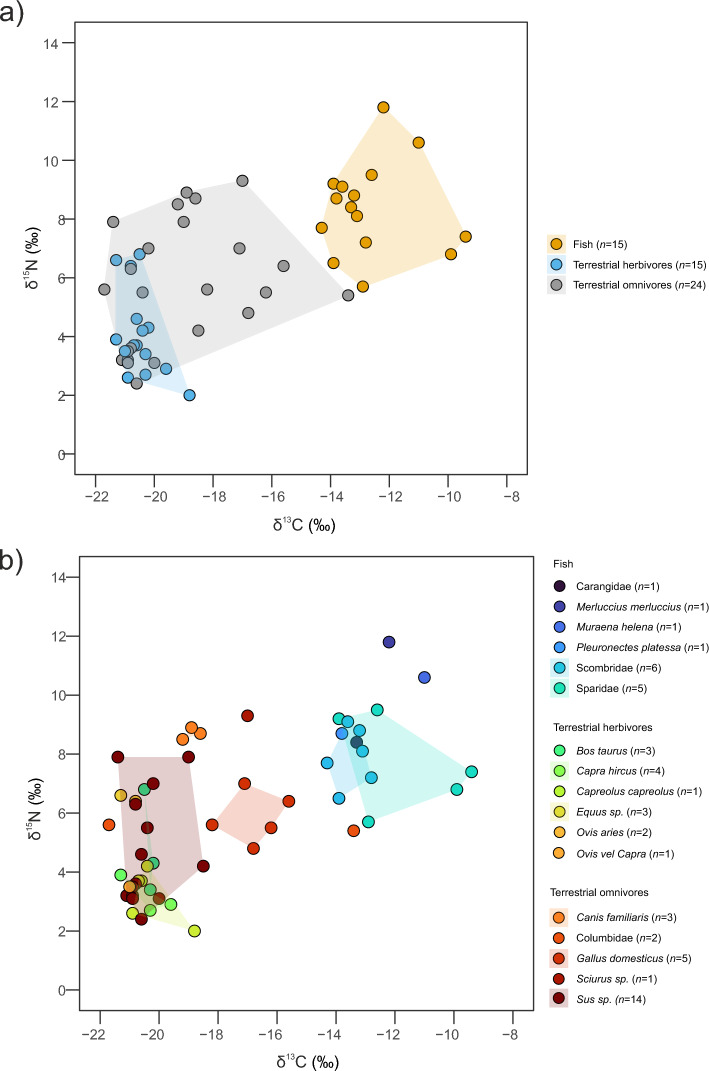
δ^13^C and δ^15^N values of terrestrial animals and fish from Pompeii analysed in this study. Polygons represent the convex hull areas that cover the distribution of values of each category. (**a**) the faunal assemblage is divided in three main categories (terrestrial herbivores, terrestrial omnivores and fish); (**b**) focus on the faunal assemblage with categorisation according to species, genus or family.

First, we categorised our samples into three broad groups: terrestrial herbivores, terrestrial omnivores, and fish. These groups are distinguishable based on their δ^13^C and δ^15^N values (Table 1; Fig. 3a). Terrestrial herbivores show δ^13^C values characteristic of a C_3_ diet, with a mean of –20.5‰ [CI: −20.8, −20.1] and relatively low variability. On average, the terrestrial omnivore group is more ^13^C-enriched compared to terrestrial herbivores (–19.1‰ [CI: −20.0, −18.2]), although their diets remain predominantly C₃-based, with some exceptions. This difference is not statistically significant (Wilcoxon rank-sum test: W = 119.5, p-value = 0.08292), but the ~ 1.4‰ enrichment in mean δ^13^C values may suggest a certain contribution from C₄ resources. δ^15^N values of terrestrial omnivores are significantly higher than those of terrestrial herbivores, with means of 5.7‰ [CI: 4.8, 6.6] and 4.1‰ [CI: 3.3, 4.9], respectively (Student’s *t*-test: t = −2.5647, df = 37, p-value = 0.01452), consistent with higher trophic level. The fish group is clearly separated from terrestrial animals, showing higher δ^13^C (–12.7‰ [CI: −13.5, −11.8]) and δ^15^N values (8.4‰ [CI: 7.5, 9.2]); these differences are statistically significant compared to terrestrial animals (Wilcoxon rank-sum test: W = 5, p-value = 0.00000002939 for carbon and W = 64, p-value = 0.00001062 for nitrogen). When the faunal assemblage is divided according to more specific categorisations, distinct patterns emerge.

### Terrestrial herbivores

The three cattle samples present consistent stable carbon isotope values but divergent stable nitrogen isotope values (Table 1;Fig. 3b), indicating a mix of practices in the management of cattle in Pompeii. Varro reports that cattle were not only left to graze freely but also supplemented with various plants (*Rust*. 2). Four goat samples were analysed, showing consistent δ^13^C and δ^15^N values (Table 1;Fig. 3b), lower than those of sheep and cattle, which may point to browsing habits and greater consumption of ligneous plants. The similarity of these values with those of the only wild terrestrial herbivore in this study (PSSG3—*Capreolus capreolus*, roe deer) suggests that these species occupied similar isotopic niches (Supplementary Table 1;Fig. 3b). Sheep show δ^13^C values similar to goats but higher δ^15^N values (Table 1;Fig. 3b). Indeed, Varro notes that sheep were allowed to graze on cultivated fields with the secondary purpose of preparing the soil for the next year’s harvest and that the diet of sheep often included supplements, such as hay, bran, grape seeds and fig leaves (*Rust.* 2.2). Sheep may have been pastured at higher densities or integrated in arable agriculture differently than goats. The equid samples exhibit the lowest mean δ^15^N values of the entire animal assemblage (Table 1;Fig. 3b). Columella refers that equids should graze freely but also be supplemented with cereals and legumes (*Rust.* VI, 27). This mixed cereal-legume diet aligns with the isotopic evidence from our study and with the composition of fodder collected from the Pompeian stables^31^.

### Terrestrial omnivores

The most abundant group comprises pigs (n = 14). Wild and domestic pigs are challenging to separate, especially in small samples where population-levels cannot be applied^32^. Considering that the vast majority of *Sus* bones identified at Pompeii in past studies derive from domestic animals, the modestly sized animals sampled in this study are also expected to derive from domestic pigs. While this group shows little variation in stable carbon isotope values, there is considerable variability in stable nitrogen isotope values, demonstrating isotopically diverse diets and potentially levels of omnivory (Table 1;Fig. 3b). Previous research indicated an herbivorous diet for pigs from *Portus Romae* and the rural site of Vagnari in Roman Puglia^33,34^. However, this may not be the case for all pigs in our study. Pigs are highly adaptable animals, as noted by Columella, who suggests they can thrive in forests, feeding on various fruits year-round, or graze in meadows, particularly marshy areas (*Rust.* 7.9), where they can access roots and insects like worms. Notably, the vicinity of Pompeii was abundant in marshes^6^. It is also possible that pigs with higher δ^15^N values were fed with human food remains or by-products from food processing. The separate and relatively consistent stable isotope values measured in chickens are distinct from other livestock. Compared to other terrestrial animals from Pompeii, and a large sample of domestic fowl from prehistoric Europe^35^, chicken samples exhibit more ^13^C-enriched δ^13^C values indicative of C_4_ plant consumption or marine products (Table 1; Fig. 3b). Chickens therefore appear to have been fed a distinct diet rather than solely C_3_ crops and processing by-products or table scraps, suggesting a somewhat separate production regime. These results align with historical testament for a preference for “millet” feed in chicken husbandry^36^. The similarity in the dietary regimes of chickens is evident across the town, as samples derived from two distinct locations: Porta Stabia and Vicolo di Cecilio Giocondo. Furthermore, we analysed two samples belonging to the Columbidae family. One (PACo1) was confidently identified as a pigeon (*Columba livia/oenas*) and it exhibits isotopic values typical of a C_3_-based diet (δ^13^C = −21.7 ‰, δ^15^N = 5.6 ‰). The second sample has a clear C_4_-based diet (δ^13^C = −13.4 ‰, δ^15^N = 5.4 ‰). The utilisation of C_4_ crops’ feed appears therefore confined to birds in Pompeii, which suggests that they may have been managed through a separate feeding regime from other domestic animals, possibly reflecting a distinct husbandry or provisioning system within the broader urban food economy (Fig. 3b). Three samples of domestic dogs show consistent isotopic values (Table 1; Fig. 3b), indicative of an omnivorous diet of the C_3_ type. Finally, one sample identified as a squirrel (*Sciurus* sp., PSSQ1) has an omnivorous diet (δ^15^N = 9.3) with a mixed C_3_-C_4_/marine signal (δ^13^C = −17) (Fig. 3b).

### Fish

The information regarding the diet and behaviours of the fish analysed in this study is sourced from FishBase^37^. Considerable variability is observed within this group (Table 1;Fig. 3b; Supplementary Table 1). The highest δ^15^N value is found in the European hake sample (PSM1, *Merluccius merluccius*), a marine demersal predatory fish, with δ^13^C values indicative of a marine environment. Following closely are the Mediterranean moray (*Muraena helena*), a solitary and territorial marine fish feeding on fish, crabs, and squid in rocky bottoms. The remaining fish samples show lower stable nitrogen isotope values, including the European plaice (*Pleuronectes platessa*), a demersal fish which primarily feeds on mollusks and polychaetes in marine, brackish, and rarely freshwater bodies. Six samples from the Scombridae family were identified, known as predators of the open sea. For example, the Atlantic bonito (*Sarda sarda*) is a neritic and epipelagic schooling fish that may enter estuaries. The Sparidae group, represented by five samples, exhibits significant variability in stable carbon and nitrogen values. These carnivorous fish are primarily marine but can also be found in brackish waters, feeding on hard-shelled benthic invertebrates. Notably, there are differences within the family; for instance, seabreams like the *Sparus aurata* are sedentary, solitary fish entering coastal lagoons and estuaries in spring. They are primarily carnivorous but occasionally herbivorous, which may explain some of the more ^13^C-depleted δ^13^C values. In this assemblage, we do not observe correlation between fish size (estimated total length) and δ^13^C and δ^15^N values (Spearman correlation: S = 176.07, p-value = 0.8539 and Pearson correlation: t = 0.5721, df = 8, p-value = 0.583, respectively). The variability observed in δ^13^C and δ^15^N values seems to suggest the exploitation of species that occupy a variety of aquatic environments and that exhibit diverse behaviours. This supports the adoption of a range of fishing and farming strategies, which is well documented in the Roman world and particularly in the Bay of Naples area^9,10^.

### Difficulties in reconstructing the human diet in Pompeii (and similar contexts) using bulk δ^13^C and δ^15^N

Stable carbon and nitrogen isotope analysis of human bone collagen is the most widely used bioarchaeological method for investigating ancient dietary practices. When combined with a comprehensive dietary baseline, it can reveal the relative proportions of different food categories. In this context, we have the unique opportunity to examine isotopic variability of different food categories that are essentially contemporaneous, providing a clear snapshot of dietary resources in a single period. In most archaeological cases in fact, materials span broad periods, but are treated as coeval, despite representing time-averaged signals (or, conversely, very short-lived deposits), while human diets may have changed significantly due for example to climatic fluctuations, changes in trade networks or shifts in crop utilisation.

**Fig. 4:**
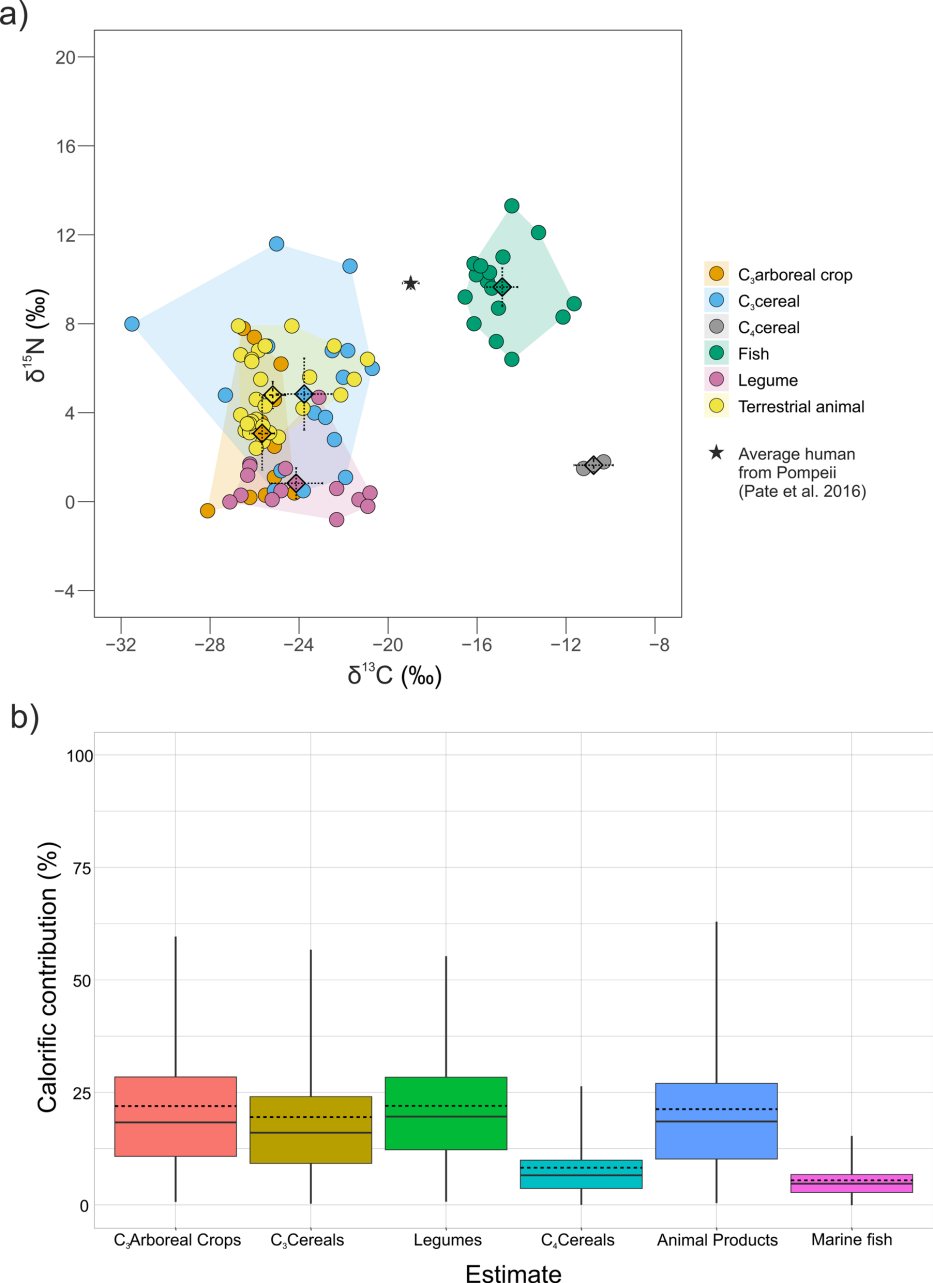
Source grouping and dietary reconstruction at 1^st^ century AD Pompeii: (**a**) δ^13^C and δ^15^N “corrected” values to represent the actual consumed food against the average human individual from Pompeii^11^. Polygons represent convex hull areas that cover the distribution of values of each category; diamonds represent mean values; dashed lines represent CIs (95%). (**b**) calorific contribution (%) of 6 food sources of an average individual from Pompeii^11^. Boxes represent 68% credible interval (corresponding to the 16th and 84th percentiles), whiskers represent 95% credible interval (corresponding to the 2.5th and 97.5th percentiles). The horizontal continuous line represents the median (50th percentile) while the horizontal discontinuous line represents the mean.

Our findings reveal a diverse range of δ^13^C and δ^15^N values across botanical and faunal samples. This leads to a critical question: can the stable isotope values of local human individuals, in conjunction with those of food categories, reveal the relative contributions of these categories to their diet? First, we need to ensure that the categories are isotopically distinguishable^38^. To do so, we must adjust the measured δ^13^C and δ^15^N values to reflect the food that was consumed. In the case of plants, we analyse the edible portion (though this is partially an assumption, especially for cereals—where we measure the grain, but what was consumed in higher quantities was likely the processed product, such as flour; we assume isotopic similarity for the purposes of this study). For animals, however, we measure bone collagen, which differs from the muscle or other tissues consumed; dietary intake likely included a mix of animal products, both primary and secondary, and fish, with variable macronutrient profiles. For this reason, we have first applied the following offsets to the faunal samples: terrestrial animals: Δ^13^C_muscle-collagen_ = −2 ‰, Δ^13^C_lipids-collagen_ = −8 ‰, Δ^15^N_muscle-collagen_ = 0 ‰; marine fish: Δ^13^C_muscle-collagen_ = −1 ‰, Δ^13^C_lipids-collagen_ = −7 ‰, Δ^15^N_muscle-collagen_ = + 1.5 ‰^12,39–42^. Following, we have calculated the “average” values of animals and fish (which includes the estimated isotopic composition from the protein fraction, i.e., muscle, and that from the lipid fraction) considering macronutrient concentrations collected from the USDA National Nutrient Database for Standard Reference expressed as dry weight (%) as reported in^12^ for each food source. As for the botanical charred material, these have been corrected to account for minimal fractionation caused by charring^43^. These values are reported in Supplementary Table 2. Faunal species that were most likely not (equids, dogs) or comparatively rarely (squirrel, columbids) consumed were not included in this analysis. We have grouped the sources as discussed earlier, avoiding species differentiation within the terrestrial animal group due to sample limitations by species. By doing so, we observe that, although some differences are detectable, the majority of food categories overlap (Fig. 4a**; **Supplementary Table 3). According to a cluster analysis performed using the NbClust() function in R (Supplementary Fig. 2a), this dataset would be in fact better represented by either two (Supplementary Fig. 2b) or three (Supplementary Fig. 2c) groups.

This is also reflected in the output of a routed and concentration-dependent mixing model using ReSources^44,45^, which evaluates the caloric contribution (%) of these food categories to the diet of an average individual from Pompeii^11^ (Fig. 4b; Supplementary Table 4; see Materials and Methods for model parameters). The results of the Bayesian mixing model suggest that C_3_ arboreal crops, C_3_ cereals, legumes and terrestrial animals each contribute broadly between ca. 0 and 50%, while C_4_ cereals and fish contribute broadly between ca. 0 and 30% each, referring to 95% credible intervals (Fig. 4b). This pattern likely reflects the model’s inability to distinguish between sources due to the substantial overlap of isotopic values among the food categories, rather than representing a real-life dietary scenario, reflecting a case of equifinality. A potential solution lies in the use of informative priors based on the available historical and archaeological evidence^46^. However, when we implemented priors in our model, the results remained largely unchanged (Supplementary Fig. 3a; Supplementary Table 5). This outcome reflects the way ReSources treats priors: as soft constraints rather than hard rules. Informative priors can only meaningfully influence the posterior distributions when the isotopic data themselves are sufficiently discriminative. In our case, the high degree of isotopic overlap between several food categories severely limited the effectiveness of priors: the model remains data-dominated, and equifinality persisted. To addresses equifinality, we have therefore grouped isotopically similar sources a posteriori^38^, sourcing from our initial cluster analysis (Supplementary Fig. 2). While this allows clearer separation between groups (Supplementary Fig. 3b; Supplementary Table 5), it reduces dietary variety to just two or three broad categories. In a context like Roman Pompeii—where material evidence points to a rich and varied diet—this necessary simplification limits the model’s ability to capture the full complexity of food consumption. Similar challenges are likely to arise in other archaeologically attested complex societies.

Beyond equifinality, it appears that additional factors may also contribute to the difficulty of reconstructing the diet of Roman Pompeians using bulk stable carbon and nitrogen isotope analysis. Specifically, it is widely recognised that the Roman diet was predominantly composed of C_3_ carbohydrates (such as cereals) and lipids (such as olive oil). Consequently, proteins derived from cereals, as a staple, likely provided a substantial portion of the amino acid requirements^47^. Therefore, any contribution from isotopically distinct protein sources, such as marine fish, would be largely masked within the overall δ^13^C values of bone collagen, as noted in previous studies^48–50^. However, it should be noted that even a seemingly minor contribution from marine sources (< 20%) could have significant economic or socio-cultural implications, necessitating its confident detection^40,51^. This is further complicated in Mediterranean contexts by the fact that fish here exhibit generally lower δ^15^N values compared to fish from the Atlantic, which can overlap with the signal of terrestrial animals and even terrestrial plants, challenging its detection relying on δ^15^N values (Fig. 4a)^50,52^.

A similar challenge has been faced while trying to evaluate the relative contribution of different food sources in the human assemblage from AD 79 Herculaneum using a Bayesian mixing model. In that case, a simple model based on just three food sources also proved inadequate. On the other hand, the Compound Specific Stable Isotope Analysis (CSIA) which uses δ^13^C and δ^15^N values of amino acids (AA) was highly effective in determining the diet of this group of humans^12^. This is because the stable isotopic signatures of bone collagen represent an average of those from single amino acids, each providing specific metabolic information^53–56^. Therefore, CSIA-AA has also the potential for the detection of additional food categories that lack protein content, such as olive oil, which are typically“invisible”in bulk stable isotope analysis. By informing a Bayesian Mixing Model of this, higher resolution estimates can be gained^12^.

## Conclusion

Our analysis of botanical and faunal remains from Pompeii and its surroundings provides new, direct insight into agricultural and animal management practices before the AD 79 eruption of Mount Vesuvius. Our isotopic data suggests varied growing conditions of C_3_ cereals and legumes, potentially including imported material, as hypothesised by scholars of the Roman economy. Differences between lentils and broad beans suggest that they were sourced and cultivated under different conditions, aligning with archaeobotanical observations while offering direct support. Stable carbon and nitrogen isotope analysis also inform on husbandry practices: pigs likely received a varied diet, while sheep and cattle were probably raised in different pastures and foddered distinctly from goats. Chickens, uniquely, display a consistent signal of C₄ plant consumption, indicating a distinct provisioning system from other livestock. Fish exhibit a wide range of δ^13^C and δ^15^N values indicative of varied aquatic environments and behaviours, consistent with archaeological and literary evidence for intensive exploitation of aquatic resources in the area.

Therefore, this study offers direct isotopic evidence that contributes to a more grounded understanding of agricultural and economic practices in Roman Pompeii. This complex system ultimately shaped the diet of Pompeii’s inhabitants. However, the diversity of available resources—with overlapping isotopic signatures—poses a challenge for reconstructing human diet using bulk stable isotope analysis, as observed using mixing models. Our study demonstrates the value of high-resolution isotopic baselines, but also exposes the limitations of bulk stable isotopes in contexts where dietary diversity mirrors economic complexity.

## Materials and methods

This project brings together Pompeian material recovered from both recent and earlier excavations campaigns with the aim to provide a more complete picture of crop and animal management from the Roman town and its surroundings. A total of 56 faunal remains and a total of 47 botanical remains were collected for the analysis from different contexts.

Plant remains were sourced from a range of locations in Pompeii and its surrounding areas, including the excavation front at the L. Frontone (2019), the bakery of Lucius Modestus, the peristyle of a house in the Insula of the Chaste Lovers (*Casti Amanti*), a garbage dump north of via di Nola in the vicolo of Cecilio Giocondo (Regio V)^57^, and the House of the Bronze Herm at Herculaneum.

Faunal remains were selected from several locations, including Insula 7 of Regio VIII in southern Pompeii near Porta Stabia^58^, the garbage dump north of via di Nola in the Vicolo of Cecilio Giocondo (Regio V)^57^, the House of Amarantus^31,59^, the House of the Garden located in Regio V, Insulae 3^31^, the Villa of the Harnessed Horse at Civita Giuliana, located approximately 700 m north of the Vesuvius Gate^31^, the Thermopolium of Insulae 3 of Regio V, the House of Castricius in Regio VII Insula 16, and the house in Regio V Insulae 6.

Stable isotope analysis of animal bones and botanical material followed standard protocols^60–63^.

Statistical tests, descriptive statistics and visualisation was performed using R version 4.4.2.

A Bayesian Mixing Model was applied using ReSources (https://isomemoapp.com/app/resources)^44,45^. δ^13^C (‰) and δ^15^N (‰) values of food sources were corrected, estimating those in the tissues consumed^12^ and associated with 1SD. Δ^15^N_collagen-diet_ was set at + 5.5 ± 0.5‰, with 100% contribution from protein; Δ^13^C_collagen-diet_ was set to + 4.8 ± 0.5‰, with 74 ± 4% contribution from protein and 26 ± 4% from lipids and carbohydrates^39^. Dry weight (%) concentrations were estimated using the USDA National Nutrient Database for Standard Reference reported by^12^ associated with standard errors. The following options were used to run the model: model type: Individual targets (no shared info); Source contribution distribution: deselected “Optimal objective prior”; Covariates model: fixed intercept (cat. vars), fixed slope (num. vars). A second model was created incorporating informative priors and user estimates a posteriori. The models are available in the Supplementary material (model.pompeii.resources and model.pompeii_priorsandestimates.resources).

Detailed information on the selected materials and methods applied is provided in the Supporting Information.

## Supplementary Information

Supplementary Information 1.

Supplementary Information 2.

Supplementary Information 3.

Supplementary Information 4.

Supplementary Information 5.

Supplementary Information 6.

Supplementary Information 7.

## Data Availability

All data generated from this study are included in this published article (and its Supplementary Information files).
