# Supplementary material for: Baseline isotopic variability in plants and animals and implications for the reconstruction of human diet in 1 st century AD Pompeii
Source: Sci Rep. 2025 Aug 3;15:28308. doi: 10.1038/s41598-025-12156-7 (PMC12319099; doi:10.1038/s41598-025-12156-7)
Supplement: Supplementary file 3 — Supplementary Information 3. [file 41598_2025_12156_MOESM3_ESM.pdf]

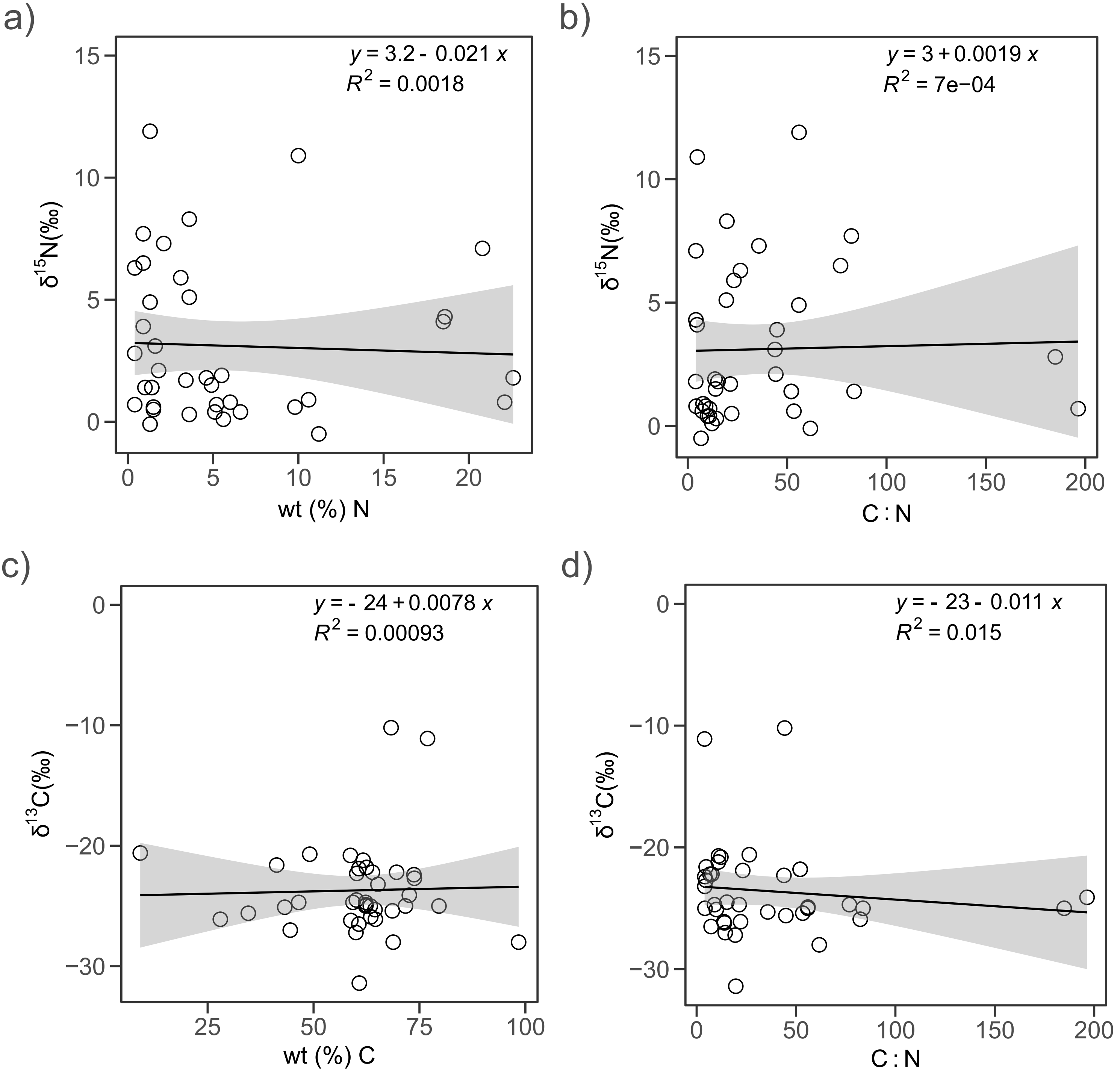

Supplementary Figure 1. Bivariate plots to explore correlation between: a) $\delta^{15}N$ (‰) and wt% N; b) $\delta^{15}N$ (‰) and C:N; c) $\delta^{13}C$ (‰) and wt% C; $\delta^{13}C$ (‰) and C:N.
