# Supplementary material for: Baseline isotopic variability in plants and animals and implications for the reconstruction of human diet in 1 st century AD Pompeii
Source: Sci Rep. 2025 Aug 3;15:28308. doi: 10.1038/s41598-025-12156-7 (PMC12319099; doi:10.1038/s41598-025-12156-7)
Supplement: Supplementary file 4 — Supplementary Information 4. [file 41598_2025_12156_MOESM4_ESM.pdf]

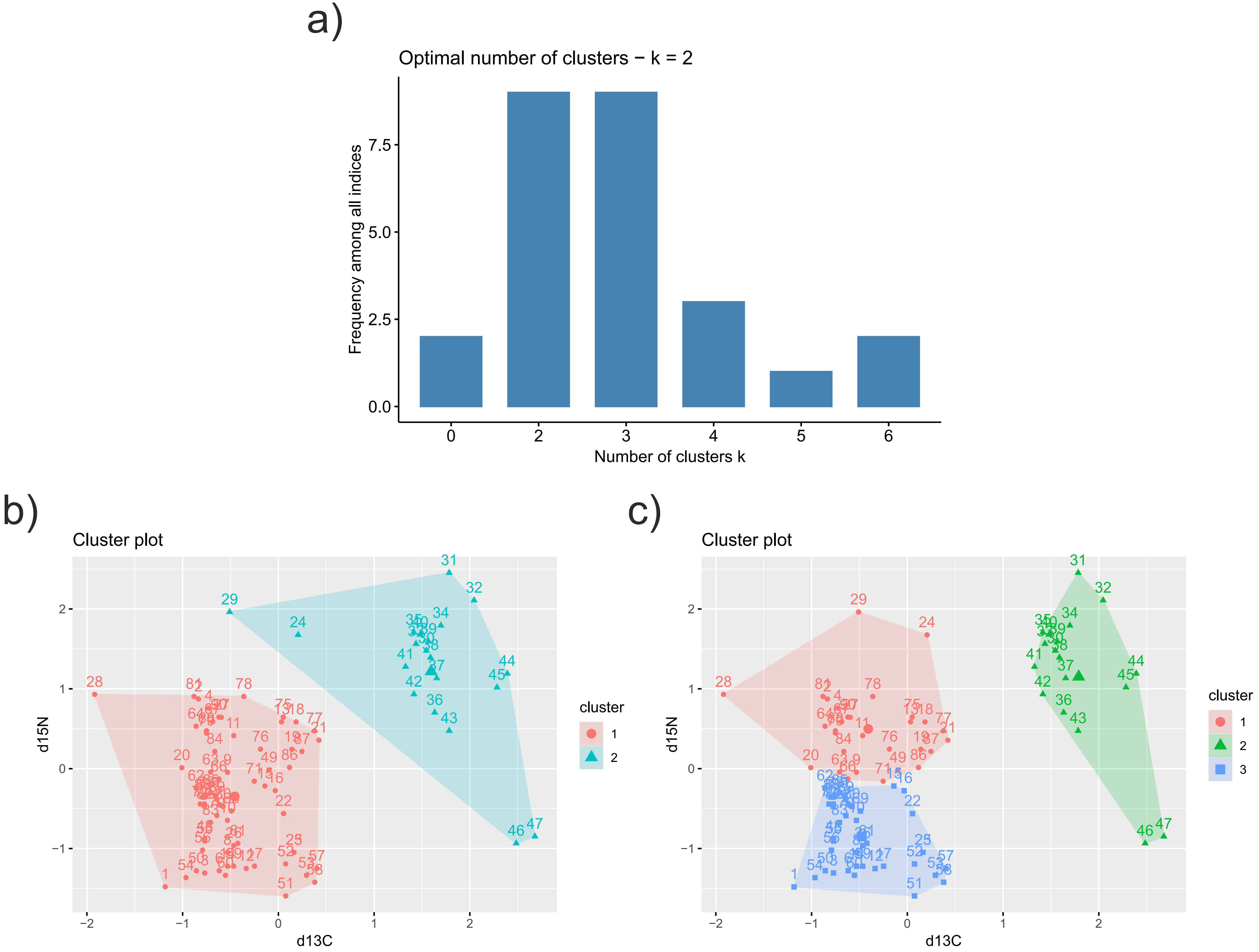

Supplementary Figure 2. Source grouping for Roman Pompeii: a) histogram showing the best clustering schemes using the NbClust() function in R; b) sources in a two-cluster scheme; c) sources in a three-cluster scheme.
