# Supplementary material for: Baseline isotopic variability in plants and animals and implications for the reconstruction of human diet in 1 st century AD Pompeii
Source: Sci Rep. 2025 Aug 3;15:28308. doi: 10.1038/s41598-025-12156-7 (PMC12319099; doi:10.1038/s41598-025-12156-7)
Supplement: Supplementary file 5 — Supplementary Information 5. [file 41598_2025_12156_MOESM5_ESM.pdf]

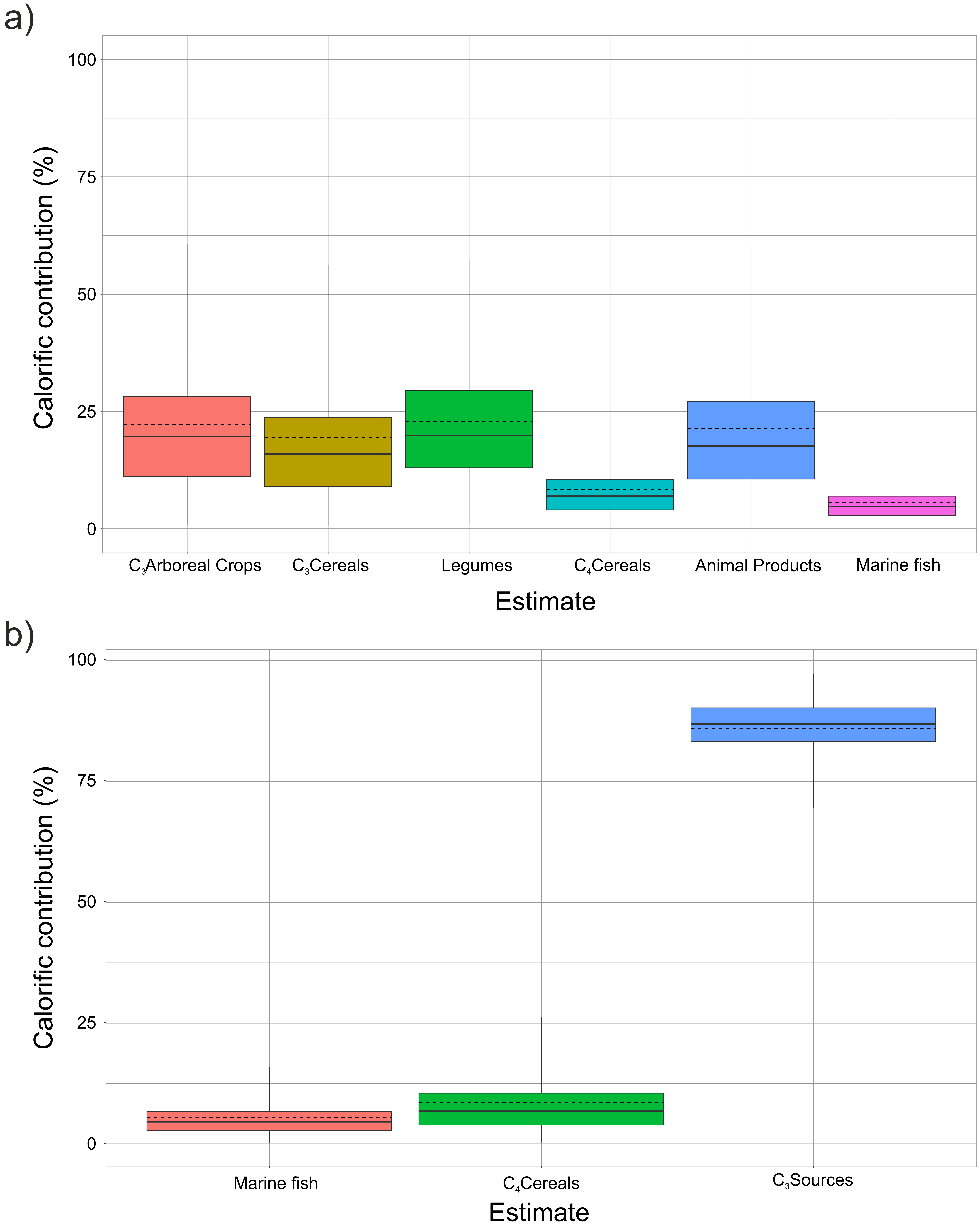

Supplementary Figure 3. Calorific contribution (%) of an average individual from Pompeii[11]: a) 6 sources using informative priors; b) 3 sources using *a posteriori* grouping. Boxes represent 68% credible interval (corresponding to the 16th and 84th percentiles), whiskers represent 95% credible interval (corresponding to the 2.5th and 97.5th percentiles). The horizontal continuous line represents the median (50th percentile) while the horizontal discontinuous line represents the mean.
