# Supplementary material for: Baseline isotopic variability in plants and animals and implications for the reconstruction of human diet in 1 st century AD Pompeii
Source: Sci Rep. 2025 Aug 3;15:28308. doi: 10.1038/s41598-025-12156-7 (PMC12319099; doi:10.1038/s41598-025-12156-7)
Supplement: Supplementary file 7 — Supplementary Information 7. [file 41598_2025_12156_MOESM7_ESM.docx]

Supporting Information

Food for Pompeii: isotopic variability in plants, animals, and implications for human dietary reconstruction at the I century AD site

Silvia Soncin, Valeria Amoretti, Chiara Comegna, Chiara Assunta Corbino, Noemi Mantile, Simona Altieri, Maria Rosa Di Cicco, Valentina Giacometti, Jan K. Bakker, Marina Caso, Angela Trentacoste, Steven Ellis, Mary Anne Tafuri, Gabriel Zuchtriegel, Oliver E. Craig & Carmine Lubritto

# Materials

This study brings together material analysed through different collaborations. Faunal material from Porta Stabia (PARP:PS) includes a broad taxonomic range: in contexts with multiple specimens, sampling prioritised species-diagnostic elements to maximize taxonomic resolution. Fish/bird remains were selected more specifically, guided by contextual factors such as deposition within single containers or size variation. Faunal material collected and studies by the Pompeii Research Laboratory derives from recent and well-defined contexts and typically represent single individuals. Botanical samples were sourced from the Pompeii Research Laboratory, the Archaeological Park of Herculaneum, and the Museo Archeologico Nazionale di Napoli. In all cases, selection criteria focused on samples with clear contextual associations and sufficient preservation for species-level identification.

Samples analysed are listed in **Supplementary Table 1** associated with contextual, archaeozoological or archaeobotanical information and stable isotope results. Below, we provide a more detailed description of the material and its archaeological provenance.

## Material from the *Pompeii Research Laboratory*

Botanical and faunal samples from the collection of the *Pompeii Research Laboratory* were selected by CC and CAC, respectively.

### Archaeobotany

Botanical material was selected based on its representation and prevalence in the Pompeian excavations, particularly focusing on species likely central to the diet of the time. Due to the destructive nature of the analysis, species expected to be part of the diet but not well-represented in the archaeological record were excluded to preserve the samples. The study exclusively examined carbonised archaeobotanical finds from various contexts dating to the 1st century AD. Some samples lacked contextual information, while others, especially those without inventory numbers, were clearly identified as being collected and analysed between 2018 and 2022.

#### Cereals

Caryopses of barley (*Hordeum vulgare* L., sample PPHV1) and wheat (*Triticum aestivum* L., sample PPTA1) were discovered in a shared pot, though the precise context is unknown due to incomplete records in the excavation diaries [^1^](https://paperpile.com/c/Ca15Ua/CKJE). These grains likely represent a preparation or storage of mixed cereals, given their co-location.

#### Legumes

The study included seeds from various legumes:

- Broad beans (*Vicia faba* L.): one sample comes from a group of seeds found inside a cooking pot (inv: 3990, rep: 823, sample PPVF1) on L. Frontone street during recent excavations (GPP). Despite the pot being located on the excavation front, it likely originated from a room in the adjacent house. The absence of fire marks on the pot suggests the seeds were possibly water-soaked before cooking (Comegna, ongoing study). A second sample (14342, LRA P/V/12, sample PPVF2) is part of a group of seeds found in room 9 of the House of the Golden Bracelet in Pompeii with no further context indication [^1^](https://paperpile.com/c/Ca15Ua/CKJE).
- Lentil (*Lens culinaris* Medik, sample PPLC1): located in a small bronze vase found on the floor of the House of the Bronze Herm at Herculaneum, possibly fallen from a shelf [^1^](https://paperpile.com/c/Ca15Ua/CKJE).
- Chickling vetch (*Lathyrus sativus* L., PPVS1) and vetch (*Vicia sativa* L., PPVS1): similar to the cereals, these seeds lack specific contextual data, originating from older excavations in Pompeii. They were part of a single archaeobotanical assemblage contained in a *caccabus*, which also included other Fabaceae species and cereals, indicating a preparation [^1^](https://paperpile.com/c/Ca15Ua/CKJE).
- Carob pods (*Ceratonia siliqua* L., sample PPCS1): Unearthed on the upper floor of a house in Herculaneum, but their exact location remains uncertain [^1,2^](https://paperpile.com/c/Ca15Ua/CKJE+Zkcah).

#### Bread

Fragments from one of the 81 loaves recovered at the bakery of Lucius Modestus in Pompeii were analysed (sample PPB2) [^1,3^](https://paperpile.com/c/Ca15Ua/CKJE+sYeQV).

#### Olive Stones

Analyses were also conducted on olive stones (*Olea europaea* L.). One sample (PPOE1) found in a refuse dump along a footpath by Cecilio Giocondo Street (Comegna, Corbino Martellone 2021). A second sample was selected (18066/B, LRA/P/V/23, sample PPOE2) from a 79 AD context without further information available [^1^](https://paperpile.com/c/Ca15Ua/CKJE).

#### Fruits

- Fig (*Ficus carica* L., sample PPFC2) and Date (*Phoenix dactylifera* L., 18083B LRAP/V/38A, sample PPPD2): found together in a Pompeian context lacking detailed excavation data [^1^](https://paperpile.com/c/Ca15Ua/CKJE). A second sample of date was selected from the same context (but with different inventory number: 18092B, LRA/P/V46, sample PPPD3) and a third one from ongoing excavation at Insula Meridionalis (sample PPPD4 from a mixture of materials used for ritual purposes dated to the Ist century AD).
- Walnuts (*Juglans regia* L., sample PPJR1): recovered from a large storage of pomegranate skins layered with straw and mats from Villa B at Oplontis (Torre Annunziata). The walnuts were likely mixed with these materials by chance [^1,2,4^](https://paperpile.com/c/Ca15Ua/CKJE+Zkcah+5Wwcn).
- Grapes (*Vitis vinifera* L., sample PPVV1): grape pips were among the few examples found in Pompeii, recently unearthed in the peristyle of a house in the Insula of the Chaste Lovers (*Casti Amanti*). They were contained in a bronze pot near a fire point, suggesting a preparation process was underway (Comegna, ongoing study).

### Archaeozoology

A total of 19 faunal samples, including bones and teeth, were selected for isotope analysis. The sampling covered two anatomical elements from pig, cattle, sheep, chicken, and pandora, likely representing two different individuals for each species. Due to the lack of other suitable elements, two samples were taken from the same anatomical element for the goat. For equids, the samples were collected from four individuals, while for dogs, they came from two. A single sample was taken from a pigeon.

All these faunal remains date back to the mid-1st century AD. The equid samples, derived from three urban and one rural individuals, perished in the 79 AD eruption. Their skeletons were discovered in various locations: the stable of the House of *Amarantus* (I, 9, 12, sample PAE1), Room 15 of the House of the Garden (V, 3, sample PAE2), the House of *Castricius* (VII, 16, 17, PAEq1), and the stable of the so-called Villa of the Harnessed Horse at Civita Giuliana, approximately 700 m north of Pompeii's Vesuvius Gate (PAE3).

The dog samples, representing pets or guard animals that also died during the eruption, were collected from the House of *Amarantus* (a male aged 5-6 years, sample PACan3) and the thermopolium of Regio V, Insula 3 (a male aged 2-4 years, PACan1).

Species such as pig, cattle, sheep, goat, chicken, pigeon, and pandora were part of the diet of Pompeii's inhabitants. Most of the food samples were retrieved from a garbage dump located north of Via di Nola, in the alley of Cecilio Giocondo. This dump, positioned between *Insulae* 6 and 7 of *Regio* V, on the sidewalk and road surface, yielded numerous pottery, glass, faunal, and botanical remains. The context suggests that these remains originated from activities in or around the alley between the earthquake of 62 AD and the eruption of 79 AD, primarily representing food waste [^5^](https://paperpile.com/c/Ca15Ua/EH4Ka).

The pandora fish remains (samples PASp1 and PASp2) were found in a storage space (V, I, 6) under the stairs of Room 25 in the House of *Leda*. This storage area served as the pantry, where an amphora (US 415, RP 515, inv. pm. 3244) filled with pandora fish was discovered. This amphora, bearing painted labels, contained 68 complete fish individuals, likely salted or preserved in brine.

## Material from the *Pompeii Archaeological Research Project: Porta Stabia* (PARP:PS)

Samples from the The *Pompeii Archaeological Research Project: Porta Stabia* (PARP:PS) were selected by AT and SE. This project brings into focus the sub-elite area of Regio I and Regio VIII in Southern Pompeii, excavating Insulae VIII.7 and I.1. The samples included in this study are from the Ist century AD levels of Insula VIII.7. This area of Pompei was densely populated with a mix of modest commercial and residential buildings, including numerous *thermopolia* and *cauponae*. The strategic position near the Porta Stabia gate likely made it a bustling entry point for goods and people entering the city [^6^](https://paperpile.com/c/Ca15Ua/qwauS).

**Property VIII.7.5**: Sample PSSC3 was selected from SU 11044, a fill from the front room of property VIII.7.5, dated to the mid-first century AD.

**Property VIII.7.9–11**: The majority of the samples (25 in total) come from SU 13034 and SU 13035, two fills from tannery vats recovered in room 56 of property VIII.7.9–11, room 56 and dated to the early first century AD. Sample PSB1 comes from SU 7037, a fill within a drain from room 44 of the same property and dated to the early 60s-79 AD.

**Property VIII.7.13–15**: Three fish samples (PST1, PSSC1 and PSSC2) were selected from SU 8008, a quarrying fill from room 78 of property VIII.7.13–15 dated to the mid-first century AD.

## Material from Museo Archeologico Nazionale di Napoli (MANN)

Thirteen botanical samples originate from an extensive and historic collection housed at the Museo Archeologico Nazionale di Napoli (MANN). This collection includes a diverse array of botanical remains recovered from ancient excavations in areas affected by the 79 AD eruption, with much of their detailed information lost over time. Samples were selected by CL.

## Material from the Archaeological Park of Herculaneum

The grain and legume samples from Herculaneum were selected by MC and sampled by SS. These include:

- Broad beans (*Vicia faba*, sample 200b): found in 1928 inside a charred basked in one of the service rooms of the *Casa del Tramezzo di Legno* III, 11-4.
- Chickpeas (*Cicer arietinum*, sample 2314c) and peas (*Pisum sativum*, sample 2317p): discovered on the upper floor of a workshop near the northeast side of the *Decumanus Maximus* during archaeological investigations in the summer of 1961. Bronze pottery, amphorae, fruits, grains, and legumes were also unearthed from the same room.
- Lentils (*Lens culinaris*, sample 692l): found inside an amphora in fragments in 1931 in the *Casa della Stoffa* IV, 19-20.
- Millet: (*Panicum miliaceum*, sample 2327m): discovered in 1961 on the mezzanine floor of one of the workshops near the northeast side of the Decumanus Maximus, together with a heterogeneous nucleus of finds, including fruits, legumes, baskets, and soles of woven rope shoes. A carbonised wooden chest containing cloth and buns also came from this room.

No archaeological contextualisation is available for barley (*Hordeum vulgare*, sample 1703b) emmer (*Triticum dicoccum*, sample 1895e) and wheat (*Triticum* sp., samples 723w and 1703w).

## Data from previous publications

The dataset analysed here accounts for stable isotope values previously published by [^7^](https://paperpile.com/c/Ca15Ua/ur6m) and [^8^](https://paperpile.com/c/Ca15Ua/LSwu). Please refer to these publications for detailed information on the material and methods used.

# Methods

## Archaeobotany

Botanical samples from the *Pompeii Research Laboratory* were identified by CC using previous [^1–4^](https://paperpile.com/c/Ca15Ua/CKJE+sYeQV+Zkcah+5Wwcn) and recent [^5^](https://paperpile.com/c/Ca15Ua/EH4Ka) studies, which also include ongoing research. For all these samples, particularly those uncovered before 2018, a comprehensive re-examination was conducted. This re-evaluation was based on the botanical atlas by Neef [^9^](https://paperpile.com/c/Ca15Ua/tCAtY) and the work of Zohary [^10^](https://paperpile.com/c/Ca15Ua/IoMfi) to ensure accurate taxonomic identification. Specifically, for grape pips, additional analyses were performed to verify their classification as cultivated varieties, referencing earlier literature [^11,12^](https://paperpile.com/c/Ca15Ua/y6P4v+DtGRm). By selecting these particular taxa, the study aimed to provide a complete overview of the plant resources available in Pompeii and its surrounding areas during the 1st century AD. This selection spans both rural and urban contexts, with a focus on sites linked to domestic activities, such as pits, latrines, and storerooms. These contexts are crucial as they reflect points of storage and consumption, offering insights into the day-to-day life and dietary practices of the Pompeian inhabitants.

## Archaeozoology

Faunal remains from the Pompeii Research Laboratory were identified by CAC. Among these, bird and mammal remains were identified using atlases [^13–16^](https://paperpile.com/c/Ca15Ua/pR0mT+QDdiU+rhDNg+BozE2) while the pandora were compared to the individuals of the reference collection of the LAZU lab of the Department of Beni Culturali at the University of Salento (Italy).

Faunal remains from Porta Stabia (PARP:PS) were identified by AT using reference material and standard zooarchaeological atlases [^13,14^](https://paperpile.com/c/Ca15Ua/pR0mT+QDdiU). Many of these elements were highly fragmented and therefore a proteomic approach was also applied (see following paragraph “Zooarchaeology by Mass Spectrometry (ZooMS)”). Further identification of fish specimens was conducted at BioArCh, Department of Archaeology, University of York, UK by JKB using the reference collection hosted there.

## Stable Isotope Analysis

### Samples from PARP:PS and the Archaeological Park of Herculaneum

Samples belonging to the PARP:PS project were analysed for stable isotopes at BioArCh, Department of Archaeology, University of York, UK by SS. Collagen was extracted following standard protocols [^17,18^](https://paperpile.com/c/Ca15Ua/FFWFZ+86nkA). Briefly, ca. 500 mg of bone were sampled from each specimen, less for fish remains. More resistant bone fragments, such as those belonging to domestic herbivores and omnivores, were mechanically cleaned from dirt and soil residues by the use of a sandblaster. Fish bones and other smaller and more fragile remains were not mechanically cleaned to avoid sample loss, also, it did not seem necessary. Terrestrial samples were demineralized at +4°C in 8 mL HCl 0.6 M solution up to two weeks, while fish samples were demineralized in a weaker acidic solution (0.1 M HCl). The acidic solution was changed every two days. When the bone fragments were flexible and translucent, the acidic solution was removed and samples rinsed three times with deionized water. 8 mL of pH3 HCl solution were added to each sample and this was heated at 80°C for 48 hrs to allow collagen gelatinization. After this time, the liquid was filtered with Ezee-filters™ in order to remove insoluble residues and subsequently with ultra-filters (Amicon® Ultra-4 Millipore™30 kDa filter centrifuge tubes) in order to analyse only molecule fractions bigger than 30 kDa. Samples were then frozen at -20°C for at least 48 hrs and freeze-dried for additional 48 hrs. Grains and legumes were washed three times with deionised water, frozen at -20°C for at least 48 hrs, freeze-dried and grounded.

Stable carbon and nitrogen isotope analysis was carried out using a Sercon 20-22 continuous flow isotope ratio mass spectrometer coupled to a Sercon GSL elemental analyzer at the BioArCh facilities, Department of Archaeology, University of York. Accuracy was determined by using standard reference materials within each analytical run. These were IAEA 600 ẟ^13^C_raw_ = -27.73 ±0.10 ‰, ẟ^13^C_true_ = -27.77 ±0.043 ‰, ẟ^15^N_raw_ = 0.93 ±0.26 ‰, ẟ^15^N_true_ = 1 ±0.20 ‰; IAEA N2 ẟ^15^N_raw_ = 20.35 ±0.15 ‰, ẟ^15^N_true_ = 20.3 ±0.20 ‰; IA Cane, ẟ^13^C_raw_ = -11.70 ±0.07 ‰; ẟ^13^C_true_ = -11.64 ±0.03 ‰. To evaluate the overall uncertainties on the measurements of each sample we relied on the method proposed by Kragten [^19^](https://paperpile.com/c/Ca15Ua/1Novu) which combines uncertainties of the international reference material and those determined from the repeated measurements of samples and reference material expressed as one standard deviation since each collagen sample was run in duplicate. The maximum uncertainty for all samples across all runs was <0.25 ‰ for ẟ^13^C and <0.32 ‰ ẟ^15^N. In addition, homogenised bovine bone collagen extracted and analysed within the same batch as the samples produced the following average values; ẟ^13^C = -22.86 ±0.07; ẟ^15^N = 6.51 ±0.19. This was within the overall mean value from 50 separate extracts of this bone sample, which produced values of ẟ^13^C = -22.96 ±0.11 and ẟ^15^N = 6.18 ±0.32.

### Samples from the *Pompeii Research Laboratory*

Samples from the Pompeii Research Laboratory were processed for stable isotope analysis at iCONa, Department of Environmental, Biological and Pharmaceutical Sciences and Technologies, University of Campania “Luigi Vanvitelli”, Italy by NM. Bone samples were mechanically cleaned using a scalpel to remove external contaminants (e.g. soil residue). Collagen extraction protocol followed a modified Longin (1971) method. Briefly, approximately 500 mg of bone fragments were demineralised at 4°C in 10 mL of HCl 0.5 M for several days. When no CO_2_ effervescence was visible and the bone texture was softer, samples were rinsed multiple times with distilled water until pH7 was reached. 10 mL of pH3 HCl solution were added to the samples, and they were heated at 70°C for 48 hours. The gelatinised collagen was filtered with an Ezee-filter™ to eliminate insoluble residues, then frozen at -20°C for 24 hours and freeze-dried for 48 hours. Collagen samples were weighed in tin capsules for EA-IRMS analysis.

Archaeobotanical samples were weighed (approximately 75 mg) and placed in glass beakers for the Acid-Alkali-Acid (AAA) pre-treatment. The protocol was adapted from well-established methods [^20,21^](https://paperpile.com/c/Ca15Ua/aPRP+9eNo). Briefly, samples were first treated with 10 mL of 0.5 M HCl for 30 minutes, or until CO_2_ effervescence ceased, to remove exogenous carbonates. The second step involved treating the samples with 10 mL of 0.1M NaOH for 15 to 30 minutes to eliminate humic acids. The final step consisted of a second acid treatment with 10 mL of 0.5M HCl for 30 minutes to remove any modern CO₂ absorbed during the alkali treatment. The entire protocol was performed at 70°C. After each step, the samples were thoroughly rinsed with distilled water until reaching a neutral pH. Dried samples were finely grounded and weighed (1.5-3.0 mg) into tin capsules.

An elemental analyzer (EA 1112 series, Thermo Scientific) and an isotope ratio mass spectrometer (IRMS Delta V Advantage, Thermo Scientific), connected in continuous flow mode (via Conflo IV, Thermo Scientific) were used for stable isotope analysis of carbon and nitrogen by SA. International certified standards were used to calibrate samples measurements: IAEA-N2 (δ^15^N= 20.3‰), IAEA-CH3 (δ^13^C= -24.724‰) and Sirfer Yeast (δ^15^N= -1.24‰, δ^13^C= -20.02‰). The aforementioned standards were included in the run list every 24 samples. The typical analytical precision, as determined from the repeated measurements of the standards, was found to be 0.1‰ for δ^13^C and 0.2‰ for δ^15^N. The same system used for the isotopic analysis was employed to simultaneously measure the concentrations of the samples. The standard Cyclohexanone 2,4 DNPH (C concentration = 51.79%, N concentration = 20.14%, Santis Analytical) was used to construct the calibration curve and to verify the accuracy of the measurements.

## Zooarchaeology by Mass Spectrometry (ZooMS)

Zooarchaeology by Mass Spectrometry (ZooMS) is based on Peptide Mass Fingerprinting (PMF) to identify species based on collagen peptides [^22^](https://paperpile.com/c/Ca15Ua/DoJC). SS applied ZooMS on faunal remains from PARP:PS. A small portion (0.3-0.7 mg) of the collagen extracted for stable isotope analysis was used for ZooMS analysis.This was dissolved in 50 μL of 50 mM ammonium bicarbonate buffer (NH_4_HCO_3_, AmBic, pH 8). Samples were digested with 1 μL of trypsin (0.4 μg/μL) and incubated overnight at 37°C. To stop trypsin activity, 1 μL of 5% v/v trifluoroacetic acid (TFA) was added to each sample. Peptide extraction was carried out using C_18_ resin ZipTip® pipette tips (Millipore), preconditioned with 200 μL of 50% acetonitrile (ACN) and 0.1% v/v TFA ("conditioning solution") and 200 μL of 0.1% v/v TFA ("washing solution"). The peptides were then eluted in 50 μL of conditioning solution. A mixture of 1 μL of eluted peptides and 1 μL of matrix solution (α-cyano-hydroxycinnamic acid) was spotted in triplicate on a stainless steel plate to be analysed via MALDI-ToF using a Bruker Ultraflex III mass spectrometer at the Centre of Excellence in Mass Spectrometry facility at the University of York. Species identification was manually conducted by examining the mass spectra for peptide m/z markers using the open-source software mMass1 [^23^](https://paperpile.com/c/Ca15Ua/0OJp). The signal-to-noise threshold was set at 3.0, and the relative intensity threshold at 0.3, using previously published markers for identification [^22,24–31^](https://paperpile.com/c/Ca15Ua/DoJC+9W9N+mtJe+GifX+Ksad+qiYZ+nZ4F+m4W1+hYRL).

1. [Borgongino, M. *Archeobotanica. Reprti Vegetali Da Pompei a Dal Territorio Vesuviano*. (L’Erma di Bretschneider, 2006).](http://paperpile.com/b/Ca15Ua/CKJE)

2. [Meyer, F. G. Carbonized food plants of Pompeii, Herculaneum, and the Villa at Torre Annunziata. *Econ. Bot.* **34**, 401–437 (1980).](http://paperpile.com/b/Ca15Ua/Zkcah)

3. [Monteix, N. *et al.* Pompéi, Pistrina recherches sur les boulangeries de l’Italie Romaine. *Mélanges l Éc. fr. Rome Antiq.* 303–306 (2011).](http://paperpile.com/b/Ca15Ua/sYeQV)

4. [Murphy, C., Thompson, G. & Fuller, D. Q. Roman food refuse: urban archaeobotany in Pompeii, Regio VI, Insula 1. *Veg. Hist. Archaeobot.* **22**, 409–419 (2013).](http://paperpile.com/b/Ca15Ua/5Wwcn)

5. [Comegna, C., Corbino, C. A. & Martellone, A. Le strade di Pompei. Le indagini archeologiche condotte nel Vicolo di Cecilio Giocondo nel 2018. in *Rivista Di Studi Pompeiani* vol. XXXII (L’erma Di Bretschneider, Roma - Bristol, 2021).](http://paperpile.com/b/Ca15Ua/EH4Ka)

6. [Ellis, S. J. R., Emmerson, A. L. C. & Dicus, K. D. *The Porta Stabia Neighborhood at Pompeii Volume I: Structure, Stratigraphy, and Space*. (OUP Oxford, 2023).](http://paperpile.com/b/Ca15Ua/qwauS)

7. [Pate, F. D., Henneberg, R. J. & Henneberg, M. Stable carbon and nitrogen isotope evidence for dietary variability at ancient Pompeii, Italy. *Mediterranean Archaeology & Archaeometry* **16**, (2016).](http://paperpile.com/b/Ca15Ua/ur6m)

8. [Soncin, S. *et al.* High-resolution dietary reconstruction of victims of the 79 CE Vesuvius eruption at Herculaneum by compound-specific isotope analysis. *Sci. Adv.* **7**, (2021).](http://paperpile.com/b/Ca15Ua/LSwu)

9. [Neef, R., Cappers, R. T. J. & Bekker, R. M. *Digital Atlas of Economic Plants in Archaeology*. (Barkhuis, Drenthe, Netherlands, 2012).](http://paperpile.com/b/Ca15Ua/tCAtY)

10. [Zohary, D., (deceased), M. H. & Weiss, E. *Domestication of Plants in the Old World: The Origin and Spread of Domesticated Plants in Southwest Asia, Europe, and the Mediterranean Basin*. (Oxford University Press, 2012).](http://paperpile.com/b/Ca15Ua/IoMfi)

11. [Stummer, A. Zur urgeschichte der Rebe und des Weinbaues. *Mitteilungen der Anthropologischen Gesellschaft in Wien* **41**, 283–296 (1911).](http://paperpile.com/b/Ca15Ua/y6P4v)

12. [Mangafa, M. & Kotsakis, K. A New Method for the Identification of Wild and Cultivated Charred Grape Seeds. *J. Archaeol. Sci.* **23**, 409–418 (1996).](http://paperpile.com/b/Ca15Ua/DtGRm)

13. [Barone, R. *Anatomia Comparata Dei Mammiferi Domestici*. vol. Osteologia (1976).](http://paperpile.com/b/Ca15Ua/pR0mT)

14. [Schmid, E. *Atlas of Animal Bones*. (Elsevier Publishing Company, 1972).](http://paperpile.com/b/Ca15Ua/QDdiU)

15. [Fick, O. K. W. Vergleichend morphologische Untersuchungen an Einzelknochen europäischer Taubenarten. *München: Ludwig-Maximilians-Universität* (1974).](http://paperpile.com/b/Ca15Ua/rhDNg)

16. [Tomek, T. & Bocheński, Z. M. *A Key for the Identification of Domestic Bird Bones in Europe: Galliformes and Columbiformes*. (Polish Academy of Sciences, 2009).](http://paperpile.com/b/Ca15Ua/BozE2)

17. [Longin, R. New method of collagen extraction for radiocarbon dating. *Nature* **230**, 241–242 (1971).](http://paperpile.com/b/Ca15Ua/FFWFZ)

18. [Brown, T. A., Nelson, D. E., Vogel, J. S. & Southon, J. R. Improved Collagen Extraction by Modified Longin Method. *Radiocarbon* **30**, 171–177 (1988).](http://paperpile.com/b/Ca15Ua/86nkA)

19. [Kragten, J. Tutorial review. Calculating standard deviations and confidence intervals with a universally applicable spreadsheet technique. *Analyst* **119**, 2161–2165 (1994).](http://paperpile.com/b/Ca15Ua/1Novu)

20. [Bogaard, A. *et al.* Crop manuring and intensive land management by Europe’s first farmers. *Proc. Natl. Acad. Sci. U. S. A.* **110**, 12589–12594 (2013).](http://paperpile.com/b/Ca15Ua/aPRP)

21. [Fraser, R. A., Bogaard, A., Schäfer, M., Arbogast, R. & Heaton, T. H. E. Integrating botanical, faunal and human stable carbon and nitrogen isotope values to reconstruct land use and palaeodiet at LBK Vaihingen an der Enz, Baden-Württemberg. *World Archaeol.* **45**, 492–517 (2013).](http://paperpile.com/b/Ca15Ua/9eNo)

22. [Buckley, M., Collins, M., Thomas-Oates, J. & Wilson, J. C. Species identification by analysis of bone collagen using matrix-assisted laser desorption/ionisation time-of-flight mass spectrometry. *Rapid Commun. Mass Spectrom.* **23**, 3843–3854 (2009).](http://paperpile.com/b/Ca15Ua/DoJC)

23. [Strohalm, M., Kavan, D., Novák, P., Volný, M. & Havlícek, V. mMass 3: a cross-platform software environment for precise analysis of mass spectrometric data. *Anal. Chem.* **82**, 4648–4651 (2010).](http://paperpile.com/b/Ca15Ua/0OJp)

24. [Buckley, M. *et al.* Distinguishing between archaeological sheep and goat bones using a single collagen peptide. *J. Archaeol. Sci.* **37**, 13–20 (2010).](http://paperpile.com/b/Ca15Ua/9W9N)

25. [Buckley, M., Harvey, V. L. & Chamberlain, A. T. Species identification and decay assessment of Late Pleistocene fragmentary vertebrate remains from Pin Hole Cave (Creswell Crags, UK) using collagen fingerprinting. *Boreas* **46**, 402–411 (2017).](http://paperpile.com/b/Ca15Ua/mtJe)

26. [Buckley, M. & Collins, M. J. Collagen survival and its use for species identification in Holocene-lower Pleistocene bone fragments from British archaeological and paleontological sites. *Antiqua* **1**, 1 (2011).](http://paperpile.com/b/Ca15Ua/GifX)

27. [Buckley, M. & Kansa, S. W. Collagen fingerprinting of archaeological bone and teeth remains from Domuztepe, South Eastern Turkey. *Archaeol. Anthropol. Sci.* **3**, 271–280 (2011).](http://paperpile.com/b/Ca15Ua/Ksad)

28. [Jensen, T. Z. T. *et al.* An integrated analysis of Maglemose bone points reframes the Early Mesolithic of Southern Scandinavia. *Sci. Rep.* **10**, 17244 (2020).](http://paperpile.com/b/Ca15Ua/qiYZ)

29. [Kirby, D. P., Buckley, M., Promise, E., Trauger, S. A. & Holdcraft, T. R. Identification of collagen-based materials in cultural heritage. *Analyst* **138**, 4849–4858 (2013).](http://paperpile.com/b/Ca15Ua/nZ4F)

30. [McGrath, K. *et al.* Identifying Archaeological Bone via Non-Destructive ZooMS and the Materiality of Symbolic Expression: Examples from Iroquoian Bone Points. *Sci. Rep.* **9**, 11027 (2019).](http://paperpile.com/b/Ca15Ua/m4W1)

31. [Welker, F. *et al.* Palaeoproteomic evidence identifies archaic hominins associated with the Châtelperronian at the Grotte du Renne. *Proc. Natl. Acad. Sci. U. S. A.* **113**, 11162–11167 (2016).](http://paperpile.com/b/Ca15Ua/hYRL)
